# Economic Burden of Hospitalization Due to Injuries in North India: A Cohort Study

**DOI:** 10.3390/ijerph13070673

**Published:** 2016-07-02

**Authors:** Shankar Prinja, Jagnoor Jagnoor, Akashdeep Singh Chauhan, Sameer Aggarwal, Ha Nguyen, Rebecca Ivers

**Affiliations:** 1 School of Public Health, Post Graduate Institute of Medical Education and Research, Chandigarh 160012, India; akashchauhan23@gmail.com; 2 The George Institute for Global Health, University of Sydney 2000, Australia; jjagnoor@georgeinstitute.org.au (J.J.); rivers@georgeinstitute.org.au (R.I.); 3 Department of Orthopaedics, Post Graduate Institute of Medical Education and Research, Chandigarh 160012, India; drsameer35@yahoo.co.in; 4 Health Economics and Social Policy Group, School of Health Sciences, The University of South Australia, South Australia 5000, Australia; Ha.Nguyen2@unisa.edu.au

**Keywords:** injury, out of pocket expenditure (OOP), catastrophic expenditure, distress financing

## Abstract

There is little documentation of the potential catastrophic effects of injuries on families due to out of pocket (OOP) expenditure for medical care. Patients who were admitted for at least one night in a tertiary care hospital of Chandigarh city due to injury were recruited and were followed-up at 1, 2 and 12 months after discharge to collect information on OOP expenditure. Out of the total 227 patients, 60% (137/227) had sustained road traffic injuries (RTI). The average OOP expenditure per hospitalisation and up to 12 months post discharge was USD 388 (95% CI: 332–441) and USD 1046 (95% CI: 871–1221) respectively. Mean OOP expenditure for RTI and non-RTI cases during hospitalisation was USD 400 (95% CI: 344–456) and USD 369 (95% CI: 313–425) respectively. The prevalence of catastrophic expenditure was 30%, and was significantly higher among those belonging to the lowest income quartile (OR-26.50, 95% CI: 6.70–105.07, *p*-value: <0.01) and with an inpatient stay greater than 7 days (OR-10.60, 95% CI: 4.21–26.64, *p*-value: <0.01). High OOP expenditure for treatment of injury puts a significant economic burden on families. Measures aimed at increasing public health spending for prevention of injury and providing financial risk protection are urgently required in India.

## 1. Introduction

Globally, 5.1 million people per year have been reported to die due to injury, accounting for around 9.2% of global mortality [1]. The magnitude and burden of injuries and violence are more devastating in developing economies, with more than 57% of the injury burden concentrated in low and middle income countries (LMICs) [1]. Among LMICs mortality due to road injuries has been projected to rise by 60% from 2015 to 2030, falls by 43% and self-inflicted injuries by 23% [1].

Injuries are the second most common cause of death after 5 years of age in India [2]. As per the National Crime Records Bureau (NCRB) report, there was a 51.8% increase in unintentional injury deaths and a 23% increase in suicidal deaths from year 2002 to 2013 [3]. Moreover from an economic viewpoint, injuries are the leading cause of death in the economically productive age group of 15–29 years [4]. Further, a large population of predominantly young people along with high vehicular density make the problem of road traffic crashes even more significant in an Indian context [5].

Although injuries make a significant contribution to the burden of disease and mortality in India, little information exists about the cost of injury. Some earlier studies estimate the cost of injuries as between 0.29% and 0.69% of Gross Domestic Product (GDP) of India [6,7,8,9]. Two of these studies used rudimentary methods of cost analysis by using insurance data which omitted many components of the cost of traffic crashes, had small sample sizes, focused mainly on direct costs, and did not take into account impact on families or estimate injury burden [6,7]. The other two studies evaluated the costs by sampling a limited number of crashes from insurance companies, hospitals, and the Motor Accident Claims Tribunal [8,9]. Mohan et al. [10], by removing factual errors from the previous study [9], reported economic loss due to road traffic injuries to be around 3.2% of GDP. Reddy et al. [11] examined both direct and indirect costs related to traffic crashes based on interviews with victims and their families. However, this study shared the limitations of the previous studies in terms of lack of cost estimates related to disability, mainly due to the cross-sectional nature of the study without consideration to future follow-ups. One study from the south Indian city of Bangalore provides estimates of OOP expenditure at the time of hospitalisation and during follow ups and reported median OOP expenditure incurred due to injuries as USD 446.5 [12]. Further, a systematic review in the context of LMICs reported the mean cost of injury ranging from U.S.$14 to U.S.$17,400 [13].

Given the potentially catastrophic effects of injuries on families and communities due to medical care in LMIC settings [14], particularly those of low socioeconomic status [15], there is a need to document both direct and indirect OOP expenditure to fully understand the impact of injuries. While the outcome of injuries can be measured as mortality, morbidity, or combined mortality morbidity indices (for example disability adjusted life years, or DALYs), the provision of comprehensive cost estimates on injuries is a powerful tool for advocating injury prevention and programs [16].

In this context, we undertook the present study to assess the OOP expenditures and catastrophic health expenditures as a result of hospitalisation due to injury. Further, we assessed coping mechanisms undertaken by the households to meet these expenses.

## 2. Methodology

### 2.1. Study Settings

The present study was undertaken in Postgraduate Institute of Medical Education and Research (PGIMER), a tertiary care institution for provision of speciality services, medical education and research located in Chandigarh, India. There are 946 doctors posted at the hospital with around 1950 beds for inpatient care. In 2013, a total of 78,568 patients were admitted while 2,128,091 patients sought outpatient consultation. We recruited patients for the present study in the trauma care centre of the study hospital from April to May 2013. The trauma centre recorded 4630 hospitalisations in 2013. The catchment area of the hospital varies within in a radius of 300–350 kilometres, which includes Union Territory of Chandigarh, north Indian states of Punjab, Haryana, Himachal Pradesh, Jammu and Kashmir and north western part of Uttar Pradesh. 

### 2.2. Study Design

This is a longitudinal prospective cohort study. All patients 18 years of age or older, admitted to hospital for at least one night (>12 h) due to any injury were eligible. Eligible patients were identified by trained research staff posted in trauma center of the study hospital. Written informed consent was administered to the patient after stabilization and provision of treatment. For participants who were unable to give consent due to injury, consent was sought from the attendant caregiver. Interviews were conducted using a structured pretested questionnaire. Data was collected at 4 standardized points of time that is during hospitalisation and subsequently at 1, 2 and 12 months after discharge. During hospitalisation, the first interview collected information on socio-demographic characteristics, context and nature of injury and contact details for follow ups. Subsequently the patient/caregiver was interviewed daily till the period of hospitalisation to elicit OOP expenditure during last 24 h. Follow up telephone interviews were undertaken at 1, 2 and 12 months after discharge from the hospital. If the participant died during follow-up, his or her family was asked to complete the interview to ascertain impact of injury related mortality at 12 months. In order to determine bias due to information collected through telephone interviews, we replicated follow-up telephone interviews with face to face interviews in a randomly selected 10% of recruited patients.

For estimation of sample size, we considered measurement of prevalence of catastrophic health expenditure rate as the primary outcome. Assuming prevalence of catastrophic expenditure to be 22% from a previous study [12], with a precision of 5.5% and 95% confidence interval, a sample size of 220 was considered appropriate. Ethical approval was obtained from the Institute Ethics Committee of the Post Graduate Institute of Medical Education and Research, Chandigarh, India (Approval code: PGI/IEC/2013/1591-92; Dated: 22/01/2013). 

### 2.3. Data Collection

Detailed information during baseline interviews was collected on socio demographic characteristics (age, gender, marital status, education, occupation and income), mechanism of injury (RTI vs. non-RTI), length of stay in hospital, OOP expenditure for hospitalisation and various coping mechanisms. Mechanism of injury was based on patient/caregivers history of events in context of the injury. Information on the OOP health expenditure was elicited both for the duration of hospitalisation as well as during follow up periods.

We collected data on both direct medical and indirect OOP expenditure. Direct expenditure includes all expenditure towards doctor/hospital charges, medicines, diagnostic tests, procedure or surgery, user fees, any informal payments, transportation charges and medical care after discharge. Informal payments, which are mostly seen in the context of health systems of low and middle income countries, are in kind or in cash under the table payments made outside official channels to providers of health care to speed up the access to health services [17]. Indirect OOP expenditure includes spending on boarding/loading/food charges for carer or relatives. If the patient had taken any treatment before coming to the study hospital, OOP expenditure on account of the same was also recorded. 

### 2.4. Data Analysis

Data was analysed using SPSS version 17 (SPSS Inc., Chicago, IL, USA) [18]. Mean amount OOP expenditure incurred for the period of hospitalisation due to injury and up to 12 months post discharge was estimated. The currency used in calculation of expenditure was Indian Rupee. For international comparison, the expenditure was then converted into USD. Conversion rate of INR to USD was taken for the year 2013. We used the conversion rate of one USD equals to 57.7 Indian Rupee during the year 2013. Impact of the expenditure was also assessed in terms of catastrophic health expenditure and distress financing.

We used the definition of catastrophic health expenditure as any OOP expenditure on treatment which was more than 30% of the annual household income [19,20,21,22]. We defined distress financing (DF) as borrowing money from relatives/friends, taking out loans from banks/other lenders, or selling assets to cope with OOP expenditure on medical treatment due to injuries [19].

Multiple logistic regression analysis was performed to examine the risk of catastrophic expenditure due to hospitalisation (outcome variable) and prevalence of distress financing(outcome variable) for recruited patients with covariates (independent factors) including age, sex, and income quartile, type of injury, hospitalisation days and presence of health insurance. Covariates were included if they had been identified as important factors in previous research. Sensitivity analysis was carried out to assess the change in the prevalence of catastrophic health expenditure by the varying the cut off from 10% to 40%.

## 3. Results

### 3.1. Recruitment

**Figure 1:**
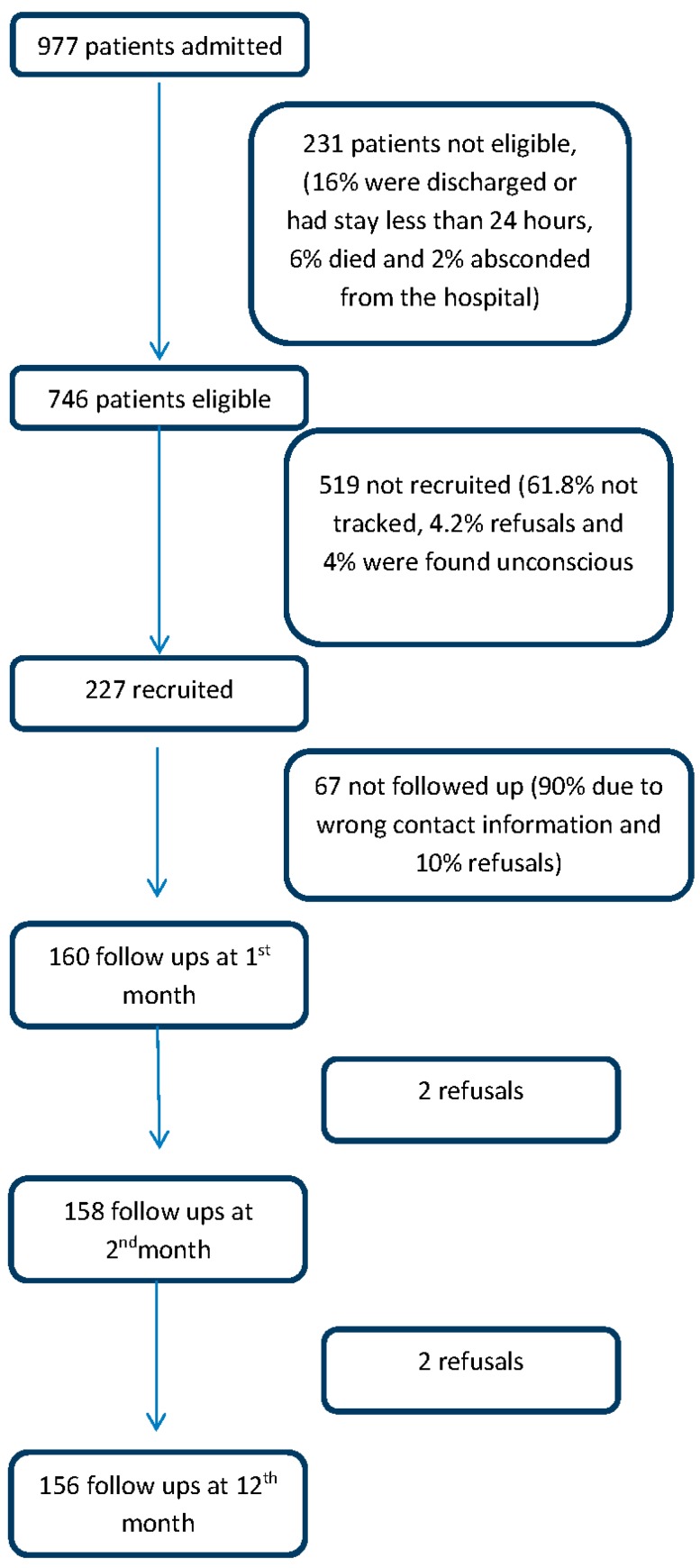
Flowchart showing recruitment procedure.

A total of 977 patients reported at the trauma centre of the study hospital during a one-month period from April to May 2013. Around 746 (76%) of them were found to be eligible; of the remaining, 16% were discharged or had a stay less than 12 h, 6.0% died and 2.0% absconded from the study hospital. Of the eligible patients, 227 (30.4%) were recruited as 62% were unable to be tracked, 4% refused to participate, and 4% were found unconscious. Around 29.5% (*n* = 67) of the patients were lost at first follow up follow up because of wrong contact information (*n* = 60) and refusals (*n* = 7). A couple of patients, during 1 month and 2 month follow up respectively, refused to participate in the interview (Figure 1).

### 3.2. Sample Characteristics

A total of 227 patients were recruited in the study. Of the total recruited patients, 60.3% (137/227) were admitted due to road traffic injuries (RTI) and the remaining 39.6% (90/227) were non-RTI patients (falls—20.0%, self-harm and assault—9.5%, burns and drowning—5.0%, collision with animate and inanimate objects—5.5%). Around 50.0% (116/227) were aged 31–60 years at the time of injury, 85.5% (194/227) were men, 24.7% (55/227) were wage labourers and around 39.6% (90/227) reported some form of health insurance at the time of hospitalisation (Table 1). Of those included, 86.7% (197/227) had sustained unintentional injuries, of which RTI constituted 70.0% (137/197). Among RTI, motorized vehicles (includes both two wheeler and four wheeler) accounted for the majority of the injuries (68.6%), followed by injuries to pedestrians (19.7%). In terms of those who underwent any surgery, around 30% underwent major surgery, 19.5% underwent minor surgery and the remaining did not had any kind of surgery.

Thirty five patients died as a result of their injuries (20 during hospitalisation, 12 at first month follow up and 3 at 12 months follow up). Further, excluding deaths, there was 30.0% loss to follow up (wrong contact information or patient not willing to participate). Excluding those who were lost to follow up, cumulative expenditure up to 12 months has been reported for 156 participants. Those who completed their 12 months follow up were compared with those who could not be followed up with. There was no significant difference reported between the two groups based on their socio-demographic characteristics, characteristics of injury, mean number of days with hospitalisation (6.5 days vs. 8 days), OOP expenditure during hospitalisation (USD 372 vs. 425) or prevalence of catastrophic health expenditure (30.0% vs. 25.0%). There was also no significant difference in OOP expenditure (USD 344 vs. USD 361), catastrophic health expenditure (26.0% vs. 26.0%) or distress financing (30.4% vs. 34.7%) among patients, when data collected using telephone interviews were compared with face-to-face interviews for follow up periods.

### 3.3. Out of Pocket Expenditure

Mean OOP expenditure per hospitalisation was USD 388 (SE-28). Specifically, average expenditure for RTI and non-RTI patients was USD 400 (SE-26) and USD 369 (SE-43) respectively (Table 2). Wage labourers reported almost 1.8 times the expenditure as compared to others. The mean OOP expenses ranged from USD 456 (SE-64) in the lowest income quartile to USD 324 (SE-41) in the richest income quartile. Expenditure was 5 times higher for patients hospitalised for more than 7 days as compared to those admitted for <3 days. Expenditure on medicine/procedure was the biggest component, constituting 75% (USD 291) of the total expenses, followed by 10% on lab diagnostics (USD 38.8), 9% on travelling (USD 35) and 6% on food (USD 23).

After 12 months, following discharge, the mean expenditure had risen by 2.5 times (USD 1046; SE-88) when compared with expenditures during hospitalisation. Specifically, expenses for RTI and non-RTI rose by 3 times and 2.3 times to USD 1179 (SE: 118) during the follow up period. Among income quartiles, expenditure pattern had reversed with highest income quartile incurring around twice the expenses as compared to poorest quartile. The detailed components of OOP expenditure are presented in Table 2.

### 3.4. Financial Risk Protection

Among 227 recruited patients, around 30% experienced catastrophic health expenditure due to hospitalisation. The prevalence of catastrophic expenditure among RTI and non RTI patients was 27.0% and 34.4% respectively. Multiple logistic regression, shown in Table 3, showed that the risk of catastrophic expenditure was inversely proportional to increasing income per capita and thus was significantly higher for those belonging to lower income quartile (OR 26.5, 95% CI: 6.70–105.07, *p*-value: <0.01), as compared to the highest income quartile. Also, those with hospitalisations of more than 7 days had higher odds of incurring catastrophic health expenditure (OR 10.06, 95% CI: 4.21–26.64, *p*-value: <0.01) as compared with those less than or equal to 3 days. The prevalence of catastrophic expenditure was 59%, 38.8% and 19.8% when the threshold for catastrophic expenditure was taken as greater than 10%, 20% and 40% of income respectively. The odds of incurring catastrophic expenditure with hospitalisation more than 7 days were statistically significant at threshold of 10%, 20% and 40% each. The bottom three income quartiles were significantly associated with the risk of catastrophic expenditure at each of the 3 thresholds of 10%, 20% and 30%. In case of application of 40% threshold, only the poorest quartile was associated with risk of incurring catastrophic expenditure. 

A total of 73 (32.15%) patients reported distress financing to cope with expenditure on hospitalisation (Table 4). The risk factors for distress financing were the same as catastrophic expenditure. The odds of incurring distress financing was 2.5 times as high for patients belonging to poorest income quartile (OR 2.50, 95% CI: 1.05–5.93, *p*-value: <0.04) as compared to richest quartile. The risk also increased with an increase in hospitalisation days, and those with >7 inpatient days showed higher odds for distress financing as compared with less than or equal to 3 days (OR 2.03, 95% CI: 0.98–4.19, *p*-value: <0.05). 

Presence of any kind of health insurance did not alter the risk for either catastrophic expenditure (OR-1.04, 95% CI: 0.89–2.24, *p*-value: 0.90) or distress financing (OR-1.12, 95% CI: 0.59–2.09, *p*-value: 0.74).

## 4. Discussion

The rising injury burden in India and other LMICs creates a greater need for generation of evidence on the economic and social costs of injury in order to allow prioritisation of prevention efforts. We undertook this study to assess the economic burden in terms of out of pocket (OOP) expenditure, catastrophic health expenditure and distress financing associated with injury for hospitalisation. Overall we found that the mean OOP expenditure due to hospitalisation was USD 388 (95% CI: 332–441), which varied from USD 400 (95% CI: 344–456) in case of RTIs to USD 369 (95% CI: 313–425) for non RTIs. The prevalence of catastrophic expenditure and distress financing was 30% and 32.1% respectively. The odds of incurring catastrophic expenditure and distress financing were greater for patients with an inpatient stay greater than 7 days and for those in the lowest income quartile.

### 4.1. Strengths 

We used a longitudinal prospective cohort study design with multiple follow up periods. Most previous studies used cross sectional design interviewing patients recall OOP expenditure on injury with a relatively lengthy recall period ranging from 15 to 365 days. This can be a major limitation when associated with expenditure data. We interviewed patients on a daily basis during hospitalisation to report OOP expenditure during last 24 h, till the discharge or death of the patient. Secondly, we also recorded the OOP expenditure on three occasions until one year post discharge from hospital. Globally, the reference period for eliciting OOP spending on rare and important events such as hospitalisation in various consumption expenditure surveys such as Living Standards Surveys is one year [23]. Similarly for out-patient consultation expenditure, a reference period of 15 days to one month is followed [23]. In India, the National Sample Surveys capture the OOP spending for any hospitalisation over the last 365 days and outpatient visits during the last 15 days [24]. In our study, in order to elicit information on OOP expenditures due to hospitalization, a recall period of 24 h was used during hospitalisation, one month during the first and second follow-up interview, and 10 months for the last follow-up using telephonic interview. Since, we use a shorter recall period for reporting OOP expenditure, we expect better recall for these periods.

Finally, in 10% of randomly selected patients who were recruited at the time of hospitalisation, a face to face follow up interview was conducted by visiting the home of the patients. This was done to determine the reliability of data collected through telephone interview. We found no significant difference in the OOP expenditures, catastrophic health expenditures or coping mechanisms on comparing data collected by two interview methods.

### 4.2. Limitations

We acknowledge certain limitations of the study. The information system of the study hospital on the intra-hospital transfer process was not very systematic. As a result some of the patients who were admitted during night to the trauma centre but referred to other wards did not have complete information on their records and thus could not be tracked. It is important to compare characteristics of those who were missed for enrolment as against those who were recruited, in order to comment on selection bias. While we compared the characteristics of those who were recruited with those who were lost to follow-up, we did not have any data on patients who were referred and could not be contacted even once. In order to address this gap, we compared the “average length of stay” and “proportion of patients who underwent for surgery”—both indicators of patient severity, between the patients who were recruited and the overall for trauma patients during the year 2013. The average length of stay and proportion who patients who underwent a surgery was 6.9 days and 30% among those who were recruited respectively and 6.55 days with 33% among all patients who underwent treatment in the trauma centre during 2013. Since the severity of the patient’s situation is an important determinant of out-of-pocket expenditures, it can be safely concluded that the expenditures reported based on the sample of patients recruited in the study represent the overall population of patients for the year 2013, i.e., there is no selection bias as a result of loss of eligible patients before recruitment.

Further, nearly 30% of the patients could not be interviewed at 12 months for collection of follow up data on OOP expenditure. However, we compared the outcomes at discharge from hospitalisation, i.e., mean OOP expenditure, catastrophic expenditure and distress financing among those who could be followed up at 12 months versus those lost to follow up, and there was no significant difference, suggesting limited possibility for selection bias. In the present study assessment of severity of injuries was not done. Assessment of injury severity requires application of Abbreviated injury scale (AIS) [25], which in turn requires clinical expertise and judgement. However, the investigators who were involved in patient recruitment did not possess medical background and thus we could not capture information on severity. Further, since recording AIS scoring was not part of routine clinical practice, the data on the same could not be extracted from the records of trauma centre. However, we do present data on length of stay and proportion of patients who underwent a surgery to represent severity.

We estimated economic burden in terms of catastrophic expenditure at 30% threshold of annual household income. While calculating catastrophic expenditure, the standard approach is to express OOPE as a proportion of a household’s capacity-to-pay, which is typically represented by non-food consumption expenditure. Here, we have used OOPE as a proportion of the annual household income for computing catastrophic expenditure. Since, the poor have a higher proportion of their overall expenditure spent on food, the non-food component of total consumption expenditure is proportionately lesser as compared to the rich. In view of this, the prevalence of catastrophic expenditure, computed using consumption expenditure as compared to income, is likely to be even higher for the poorest quintiles. This implies that the inequitable nature of OOP financing for health care is likely to be even more than what is reflected in our findings. Secondly, there are numerous critiques of the use of these thresholds to estimate financial risk protection. The criticism revolves around the use of threshold for catastrophic health expenditure relative to household ability to pay as reflected by consumption expenditure or income. The threshold varies from 10%–40% depending on the disaggregation of data for catastrophic expenditure. In our case we use the 30% threshold as the data on income were used to assess the ability to pay. This has been used by several other studies. Some authors have also proposed multidimensional indicators to assess financial risk protection [26,27]. However, this is beyond the scope of the present study. Our findings on similar prevalence of catastrophic expenditure and distress financing reflect on satisfactory performance of each indicator to measure financial risk protection. We could not offer interpretations on why insurance did not had any effect on reduction of out of pocket expenditure, as the data on the type of insurance were not collected, which could have revealed whether the trauma care was covered under the insurance, and if it was covered, whether it was appropriate in terms of benefit package rates.

### 4.3. Findings in Context with Country and Global Research

We found the average OOP expenditure per hospitalisation due to injury to be INR 22,385 or USD 388. A previous estimate based on analysis of National Sample Survey 60th round data (2004–2005) reported OOP expenditure at around one -fourth of the expenditure (USD 89) reported in the present study [28]. Another population-based survey from South India in 2004, reported OOP expenditure of INR 18,000 (USD 300) on medical treatment for RTI [29]. This difference in OOP expenditure could be as a result of the household survey based data which includes whole severity range of injury patients. On the other hand our study was undertaken in a tertiary care setting hospital reporting mostly severe cases. Another reason for increase in the expenditure over the time period of 2004–2005 to 2103 may either be due to the increase in medical cost over the years (taking into consideration the inflation rate) or increase in utilization of health care because of increase in awareness and demand among patients. Further, a systematic review examining the cost of injuries in LMICs found mean expenditure in the range of USD 14 to 17,400 [13]. Specifically, for RTIs, studies done in other South East Asia Region (SEAR) countries such as in Pakistan [30], and Vietnam [31], reported OOP expenditure in the range of USD 355–363. 

The average annual household income in the study population was around INR 0.16 million (USD 2738). When compared with the average cost of injury for a year (INR 60,367 or USD 1046), it was seen that a significant proportion, (40%) of this income was utilized in the medical treatment of the injury. Research shows that individuals belonging to the lowest income groups live much closer to the household’s financial survival threshold (household’s costs of basic necessities) [32,33], and our study supports these findings. For the poorest 25% population, nearly 73% of annual income was utilized to pay for treatment costs, compared to 20.5% among the richest quartile.

Monetary indicators such as indirect costs or income losses due to injury, which may have contributed to the financial burden, could not be accurately in the present study. Around 92% of the injured patients were from the economically productive age group of 18–60 years, out of which 76% of the patients were actually an earning member of the family. Further among lower income quartiles, around 57% of the patients were either wage labourers or cultivators, as compared to 53% of the patients in the upper incomes quartiles working as an employee or as a regular salaried employer. Considering this, it could be concluded that injury actually resulted in indirect income loss among families especially among those who belonged to the lower income quartiles as these people were depended on the daily wage.

The 30% prevalence of catastrophic expenditure found in our study is comparable to 20% prevalence found in another Indian based study done in similar urban settings [12]. Findings from a SEAR countries of Vietnam showed a similar 26% prevalence of catastrophic expenditure [31]. Comparing injuries with other conditions, data from the NSS, India, shows that hospitalisation with injuries results in 22% higher odds of incurring catastrophic spending as compared to hospitalisation due to any communicable diseases, showing expensive nature of the treatment of injuries [28]. Because of the high severity of injuries, immediate medical attention is required, which further necessitates the need of immediate money in the hands of households, forcing them to undertake risky sources like borrowings or selling of assets, leaving them vulnerable to impoverishment.

In the present study, 34% of the patients reported distress financing due to injuries, and furthermore this prevalence was significantly higher in the poorest income group as compared to the richest. A study done in similar settings [12] found 69% of affected households affected by these financing mechanisms, and as per NSS, India, 2004–2005 [28], distress financing was in the range of 39%–45% due to injuries. An Indian population based survey on road traffic injuries documented that 66% of the poor households had to borrow money and 21% had to sell assets to cope up with the expenditure [29]. Thus, these previous studies report a higher prevalence of distress financing than our study. This could be a result of enrolment from public and private hospitals whereas the patient sample in our study was from a public hospital. In the context of India, whole of the treatment expenses are paid out of pocket for getting treatment in private hospitals, where as in public hospitals, treatment is subsidised by the government and thus only some of the proportion of the total expenditure is paid out of pocket. Further, even being a public hospital, patients recruited in the study still reported a significant expenditure and most of this expenditure (95%) was on drugs/procedure and diagnostics. This findings is very similar to previous studies which point out that medicines are the major cause of OOP spending in public sector hospitals in India [34,35]. There has been an initiative in terms of free drug policy, from state governments such as that of Rajasthan, Tamil Nadu and Kerala, the replication of which in other states could have far reaching impact. Drug procurement models in these states in terms of mixed procurement system i.e., centralized procurement and distribution have resulted in lowering of purchasing price, better availability and utilization, and reduction in OOP expenditure [36]. When compared with other illnesses, distress financing ranged from 38% to 42.6% in case of heart disease and cancer respectively [28].

## 5. Conclusions 

The present study showed that a significant amount of OOP expenditure was incurred by the households getting treatment for injury in a public sector tertiary care hospital in India. Although treatment in subsidized in public sector hospitals in India, a significant amount of expenditure was still incurred for the drugs/procedure and diagnostics. Due to this expenditure, around one third of the households had fallen into the trap of catastrophic health expenditure and distress financing.

Our findings have some important policy implications. Firstly, there is a need to increase the extent of financial risk protection for injury related medical care. The Government of India is in the process of developing a Universal Health Care Package program. High rates of catastrophic health expenditure on account of injury imply that injury should be part of any benefit package for universal health care in India. Numerous central and state publicly financed health insurance schemes have been initiated in India [37]. Care for injury patients should be a part of their benefit package. The height of the benefit package, i.e., the level of financial protection as percentage of total health care costs, should be appropriately designed to provide adequate financial risk protection. Secondly, the study also supports the development of upstream or preventive measures in the terms of laws such as compulsory motorcycle helmets, seat belts, child restraints and enforcement of traffic rules by the concerned authorities strictly. As seat-belt wearing rates among drivers is around 27% [38] and that of motorcyclists wearing helmets is around 23% [39] in India, there is a need to educate the public and create awareness for the greater uptake of these preventive measures, and strengthen regulatory measures to enforce its practice. Further, on the part of government, there is a need for provision of well-constructed roads along with separate lanes for slow-moving and fast-moving vehicles and proper footpaths for pedestrians. Study from India shows that out of the total expenditure incurred on RTIs, around 1% constituted medical expenditure and rest is due to productivity losses [11]. Further, when this whole expenditure is added up it amounts to 3.2% of the GDP of India [10]. If the preventive measures above are adopted, not only would it lead to the reduction in the occurrence of out of pocket expenditure, but the economy would also gain from the reduction in productivity losses.

Finally, our results also strongly suggest that poorest are the hardest hit by the economic burden of financing healthcare for injury. As a result, it appears to pose a financial barrier to access. The OOP expenditure at hospitalisation for the poorest is higher than the wealthiest, which reverses at 12 month post discharge. Given that there is no difference in injury severity, this implies that either the poor are foregoing necessary care post discharge, or they are substituting it with inferior and cheap alternatives; that is an inequitable impact from the economic burden of injury on access to care. Together with vertical inequity in financing and poor financial risk protection for poor, it implies that there can be no universal health coverage without adequate coverage for injury care.

## Figures and Tables

**Table 1:** Socio-demographic characteristics of hospitalised injury patients.

Variable	Category	N (%)	RTI * (%)	Non-RTI (%)
Gender	Male	194 (85.5)	119 (86.9)	75 (83.3)
	Female	33 (14.5)	18 (13.1)	15 (16.7)
Age Group	18–30 years	93 (41.0)	60 (43.8)	33 (36.7)
	31–60 years	116 (51.0)	68 (41.6)	48 (53.3)
	Above 60 years	18 (8.0)	9 (6.6)	9 (10.0)
Marital Status	Unmarried	67 (29.5)	45 (32.8)	22 (24.4)
	Married	153 (67.4)	86 (62.8)	67 (74.4)
	Other	7 (3.1)	6 (4.4)	1 (1.1)
Education	Illiterate	39 (17.2)	20 (14.6)	19 (21.1)
	Primary and middle	73 (32.2)	39 (28.5)	34 (37.8))
	Matric and above	115 (50.7)	78 (57.0)	37 (41.0)
Occupation	Cultivator	29 (12.8)	15 (10.9)	14 (15.6)
	Wage Labourer	55 (24.7)	35 (25.5)	20 (22.2)
	Employer	26 (11.5)	14 (10.2)	12 (13.3)
	Unpaid family worker	26 (11.5)	14 (10.2)	12 (13.3)
	Regular salaried	58 (25.2)	40 (29.2)	18 (20.0)
	Others	33 (14.5)	19 (13.9)	14 (15.5)
Income Quartile (INR)	Poorest (<10,000)	59 (26.0)	32 (23.4)	27 (30.0)
	Poor (10,001–18,000)	56 (24.7)	30 (21.9)	26 (28.9)
	Rich (18,001–35,250)	55 (24.2)	36 (26.3)	19 (21.1)
	Richest (35,251–552,000)	57 (25)	39 (28.5)	18 (20.0)
Health Insurance	Yes	90 (39.6)	58 (42.3)	32 (35.6)
	No	137 (60.4)	79 (57.7)	58 (64.4)
Cause of Injury	Unintentional	197 (86.7)	137 (100)	60 (66.6)
	Intentional	21 (9.2)	-	21 (23.3)
	Undetermined	9 (3.9)	-	9 (10.0)
Mode of Transportation	Pedestrian	-	27 (19.7)	-
	Bicycle	-	9 (6.5)	-
	MTV **	-	94(68.6)	-
	Others	-	7 (5.0)	-
Catchment area	Punjab	75 (33.0)	48 (35.0)	27 (30.0)
	Haryana	62 (27.0)	39 (28.5)	23 (25.6)
	Himachal Pradesh	45 (20.0)	20 (14.6)	25 (27.8)
	Chandigarh	20 (9.0)	17 (11.7)	3 (3.3)
	Jammu and Kashmir	6 (4.0)	3 (2.2)	3 (3.3)
	Others	15 (6.0)	7 (5.1)	8 (8.8)
	Not found	4 (1.7)	3 (2.0)	1 (1.1)
Total		227 (100)	137 (100)	90 (100)

* RTI: Road traffic injury, ** MTV: Includes both motorised two wheeler and four wheeler vehicles.

**Table 2:** Mean out of pocket (OOP) expenditure (USD) during hospitalisation and 12 months following injury.

Variable	Category	Mean Out of Pocket Expenditure in USD (SE)							
		During Hospitalization					12 Months Following Injury		
		RTI *	Non-RTI	*p*-Value	All Injuries	RTI *	Non-RTI	*p*-Value	All Injuries
Overall		400	369	0.58	388	1179	850	0.07	1046
		(36)	(43)	5	(28)	(118)	(136)	2	(88)
Gender	Male	402	374	0.66	391	1168	937	0.26	1080
		(40)	(53)	3	(32)	(127)	(163)	5	(100)
	Female	389	345	0.69	369	1282	484	0.01	826
		(81)	(73)	3	(54)	(296)	(104)	1	(162)
Age Group	18–30 years	332	323	0.906	329	986	616	0.089	853
		(39)	(70)		(35)	(131)	(165)		(105)
	31–60 years	459	407	0.589	438	1380	863	0.051	1150
		(63)	(68)		(46)	(207)	(134)		(132)
	Above 60 years	416	328	0.516	372	1010	1793	0.429	1289
		(88)	(98)		(65)	(265)	(1229)		(452)
Marital Status	Unmarried	307	290	0.837	301	910	758	0.572	859
		(43)	(80)		(39)	(147)	(233)		(24)
	Married	450	399	0.510	427	1323	896	0.073	1136
		(52)	(55)		(38)	(164)	(164)		(118)
	Other	403	92	0.422	358	986	92	0.287	762
		(134)	(XX) **		(122)	(311)	(XX) **		(313)
Education	Illiterate	324	494	0.256	407	801	740	0.769	774
		(58)	(138)		(74)	(106)	(186)		(100)
	Primary and middle	438	417	0.857	428	1189	829	0.243	1020
		(83)	(78)		(57)	(225)	(201)		(153)
	Matric and above	402	259	0.059	357	1284	918	0.228	1157
		(47)	(42)		(35)	(174)	(248)		(143)
Occupation	Cultivator	191	428	0.073	306	542	1354	0.068	884
		(36)	(126)		(66)	(86)	(479)		(221)
	Wage Labourer	515	441	0.663	488	1161	359	0.014	894
		(100)	(138)		(80)	(215)	(76)		(158)
	Employer	267	267	0.996	267	1170	1054	0.829	1120
		(47)	(71)		(41)	(407)	(270)		(254)
	Unpaid family worker	474	365	0.416	424	1267	524	0.036	895
		(94)	(89)		(65)	(296)	(130)		(181)
	Regular salaried	454	423	0.804	444	1583	999	0.119	1371
		(70)	(102)		(57)	(256)	(182)		(180)
	Others	287	226	0.519	261	672	915	0.747	794
		(60)	(69)		(45)	(211)	(708)		(360)
Inc. Quartile	Poorest	436	479	0.744	456	915	601	0.206	777
		(73)	(113)		(64)	(190)	(129)		(122)
	Poor	328	307	0.810	318	727	602	0.409	663
		(70)	(47)		(43)	(115)	(97)		(75)
	Rich	523	318	0.173	452	1530	1231	0.562	1458
		(96)	(92)		(71)	(240)	(505)		(217)
	Richest	314	346	0.725	324	1493	1362	0.808	1443
		(42)	(96)		(41)	(309)	(465)		(257)
Health Insurance	Yes	445	443	0.986	445	1418	1031	0.272	1287
		(60)	(99)		(52)	(220)	(230)		(166)
	No	368	328	0.542	351	1006	767	0.240	900
		(46)	(45)		(32)	(123)	(166)		(101)
Hospitalised days	<3 days	174	113	0.080	150	618	531	0.630	579
		(26)	(14)		(17)	(89)	(159)		(85)
	4–7 days	377	385	0.923	380	1342	957	0.319	1198
		(33)	(88)		(40)	(216)	(338)		(185)
	>7 days	784	712	0.606	755	1734	1282	0.210	1562
		(97)	(89)		(68)	(257)	(165)		(173)

* RTI: Road traffic injury, ** single case was reported in this category, SE: Standard error.

**Table 3:** Prevalence of catastrophic expenditure during hospitalization and its risk factors.

Variable	Category	Number with of Catastrophic Expenditure (%)	OR *	95% CI **	*p*-Value
Sex	Male	58 (29.8)	1.40	0.50–3.92	0.51
	Female	10 (30.3)	1		
Age	18–30 years	20 (21.5)	1		
	31–60 years	42 (36.2)	1.93	0.88–2.22	0.96
	>60 years	6 (33.3)	1.63	0.42–6.39	0.47
Income Quartile	Poorest	24 (57.6)	26.50	6.70–105.07	<0.01
	Poor	19 (33.9)	12.18	3.01–49.32	<0.01
	Rich	12 (21.8)	4.80	1.17–19.62	0.03
	Richest	3 (5.3)	1		
Hospitalised days	<3 days	14 (14.6)	1		
	4–7 days	18 (25.2)	2.81	1.14–6.95	0.02
	>7 days	36 (58.0)	10.60	4.21–26.64	<0.01
Health Insurance	Yes	28 (31.1)	1		
	No	40 (29.1)	1.04	0.89–2.24	0.90
Type of Injury	RTI ^$^	37 (27.0)	0.86	0.42–1.78	0.70
	Non RTI	31 (34.4)	1		

* OR: Odds ratio, ** CI: Confidence interval, ^$^ RTI: Road traffic injuries.

**Table 4:** Prevalence of distress financing during hospitalisation and its risk factors.

Variable	Category	Number with Distress Financing (%)	OR *	95% CI **	*p*-Value
Sex	*Male*	65 (33.5)	1.70	0.69–4.18	0.24
	*Female*	8 (24.2)	1		
Age	*18-30 years*	25 (26.8)	1		
	*31–60 years*	43 (37.0)	1.50	0.79–2.84	0.21
	*>60 years*	5 (27.8)	1.03	0.31–3.39	0.96
Income Quartile	Poorest	24 (40.7)	2.50	1.05–5.93	0.04
	Poor	21 (37.5)	2.30	0.96–5.50	0.06
	Rich	16 (29.0)	1.38	0.56–3.42	0.48
	Richest	12 (21.0)	1		
Hospitalised days	<3 days	23 (26.3)	1		
	4–7 days	23 (32.0)	1.53	0.74–3.16	0.24
	>7 days	26 (41.9)	2.03	0.98–4.19	0.05
HealthInsurance	Yes	29 (32.2)	1		
	No	44 (32.1)	1.12	0.59–2.09	0.74
Type of Injury	RTI ^$^	44 (32.1)	1.20	0.65–2.20	0.54
	Non RTI	29 (32.2)	1		

* OR: Odds ratio, ** CI: Confidence interval, ^$^ RTI: Road traffic injuries.

## Abbreviations

RTI	Road traffic injuries
OOP	Out of pocket
LMIC	Low and middle income countries
MTV	Motorised vehicles
NSSO	National sample survey organization
